# Fractionated stereotactic radiation therapy improves cranial neuropathies in patients with skull base meningiomas: a retrospective cohort study

**DOI:** 10.1186/1748-717X-7-225

**Published:** 2012-12-28

**Authors:** Xinglei Shen, David W Andrews, Robert C Sergott, James J Evans, Walter J Curran, Mitchell Machtay, Ruben Fragoso, Harriet Eldredge, Colin E Champ, Matthew Witek, Mark V Mishra, Adam P Dicker, Maria Werner-Wasik

**Affiliations:** 1 Department of Radiation Oncology, Kimmel Cancer Center, Jefferson Medical College of Thomas Jefferson University, Philadelphia, PA, USA; 2 Department of Neurologic Surgery, Thomas Jefferson University, 909 Walnut St, 3rd Floor, Philadelphia, PA, 19107, USA; 3 Neuro-ophthalmology Service, Wills Eye Hospital, Thomas Jefferson University, 840 Waltnut St., Suite 930, Philadelphia, PA, 19107, USA; 4 Department of Radiation Oncology, Winship Cancer Institute, Emory University, 1365 Clifton Road Northeast, Atlanta, GA, 30322, USA; 5 Department of Radiation Oncology, University Hospitals, Case Western Reserve University, 11100 Euclid Ave, Cleveland, OH, 44106, USA; 6 Department of Radiation Oncology, University of California–UC Davis Cancer Center, 4501 X Street, Suite 0140, Sacramento, CA, 95817, USA; 7 Department of Radiation Oncology, University of Kansas Medical Center, 3901 Rainbow Blvd, Mail Stop 4033, Kansas City, KS, 66160, USA; 8 Department of Radiation Oncology, Thomas Jefferson University Hospital, Bodine Cancer Center, 111 S. 11th Street, Philadelphia, PA, 19107, USA

**Keywords:** Fractionated stereotactic radiation therapy, Meningioma, Symptom, Outcome, Skull base

## Abstract

**Background:**

Skull base meningiomas commonly present with cranial neuropathies. Fractionated stereotactic radiation therapy (FSRT) has been used to treat these tumors with excellent local control, but rates of improvement in cranial neuropathies have not been well defined. We review the experience at Thomas Jefferson University using FSRT in the management of these patients with a focus on symptom outcomes.

**Methods:**

We identified 225 cases of skull base meningiomas treated with FSRT at Thomas Jefferson University from 1994 through 2009. The target volume was the enhancing tumor, treated to a standard prescription dose of 54 Gy. Symptoms at the time of RT were classified based on the cranial nerve affected. Logistic regression was performed to determine predictors of symptom improvement after FSRT.

**Results:**

The median follow-up time was 4.4 years. In 92% of cases, patients were symptomatic at the time of RT; the most common were impaired visual field/acuity (58%) or extraocular movements (34%). After FSRT, durable improvement of at least one symptom occurred in 57% of cases, including 40% of visual acuity/visual field deficits, and 40% of diplopia/ptosis deficits. Of all symptomatic patients, 27% experienced improvement of at least one symptom within 2 months of the end of RT.

**Conclusions:**

FSRT is very effective in achieving improvement of cranial neuropathies from skull base meningiomas, particularly visual symptoms. Over half of treated patients experience a durable improvement of at least one symptom, frequently within 2 months from the end of RT.

## Background

Meningiomas are benign tumors which account for approximately 25% of primary intracranial tumors [1]. A significant proportion arises in the skull base region, where they are often difficult to access surgically and frequently cause cranial neuropathies. Definitive treatment recommendations for these tumors include radiation therapy (RT) and surgery (with post-operative RT after subtotal resection) [2,3]. Following complete or subtotal resection without RT, 5 year progression free survival ranges from 81-95% [2-4].

RT can provide excellent local control in the definitive and post-operative settings. Single institution series of fractionated RT using either 3-D conformal RT [5,6], intensity modulated radiation therapy (IMRT) [7], or fractionated stereotactic radiation therapy (FSRT) [8-10] have reported local control rates of 90-100%. Stereotactic radiosurgery (SRS) with Gamma Knife or linear accelerator (LINAC) based SRS have also resulted in excellent local control [11-13].

Although local control data have been extensively described, most series fail to evaluate symptomatic outcomes for these patients. An overview of published literature indicated that 14-44% of patients have clinical neurological improvement after FSRT, and up to 95% have stabilization of neurologic symptoms [14]. However, a paucity of literature exists with regards to details of symptom outcomes, such as timing of symptom improvement, durability of symptom improvement, and prognostic factors.

At our institution, we have treated skull base meningiomas both definitively and post-operatively with FSRT since 1994. Our institutional philosophy has been to preferentially treat skull base meningiomas with FSRT instead of SRS based on the theoretical radiobiological advantage of fractionation to achieve greater sparing and recovery of sensitive normal structures such as the optic apparatus. We have observed an unexpected rate of durable symptomatic improvement during and soon after FSRT treatment. This report will review our experience of treating patients with skull base meningiomas with FSRT, focusing our analysis and discussion on symptom outcomes.

## Methods

### Patient selection

We reviewed all patients with skull base meningiomas treated with FSRT from 1/1/1994 to 3/1/2009. Skull base meningiomas were defined as any clinically or pathologically diagnosed meningioma which involved the cavernous sinus, sphenoid, clinoid, sella, suprasellar region, cerebellopontine angle, petroclival region, foramen magnum, clivus, or designated as skull base, not otherwise specified (which tended to be large tumors encompassing multiple regions). For analysis of local control, we classified patients into four treatment categories: RT alone, adjuvant RT (after subtotal or near total resection), RT for progression/recurrence after surgery, or re-irradiation (with or without prior surgery).

### Radiation delivery

All patients were treated on a dedicated stereotactic linear accelerator (Varian SR600 or Novalis TX). Gross tumor volume (GTV) was equivalent to clinical target volume (CTV) and planning target volume (PTV), and was defined as the contrast enhancing tumor only. Our standard prescription dose was 54 Gy (range 10 – 60 Gy, median 52.2 Gy) in 1.8 or 2.0 Gy/fraction. Dose was reduced in 56% of cases when the optic chiasm dose exceeded 56–57 Gy, or in re-irradiation cases. Prior to 2004, treatment planning was performed on the Radionics XKnife (Radionics, Inc., Burlington, MA) treatment planning system, with multi-isocenter plans (median 79% isodose line) using spherical collimators and Gill-Thomas-Cosman frames for patient immobilization. After 2004, patients were planned with BrainLab (BrainLAB AG, Feldkirchen, Germany) treatment planning system with single isocenter conformal plans using 5 dynamic arcs (median 90% isodose line), and thermoplastic mask for patient immobilization with position verification using ExacTrac kV daily imaging. Static stereotactic intensity modulated radiation therapy (IMRT) was introduced in 2004, and was generally used for large tumors with eccentric shapes or those close to critical structures.

### Follow-up

Patients followed up with a radiation oncologist at 6 weeks after completion of RT. Subsequent follow-up visits with either a radiation oncologist or neurosurgeon occurred at 3 months, 6 months, and 1 year, and then yearly and bi-annually up to 10 years, and then per patient preference afterwards. Length of follow-up was defined as time from the start of RT to the last follow-up with radiation oncology or neurosurgery. Time to progression was defined as the time from start of RT to the date of first radiographic evidence of progression.

### Symptoms and toxicity

Symptoms were classified into major categories based on the cranial nerves (CN) involved: visual field/visual acuity (CN2), extra-ocular movements/ptosis (CN3, 4, 6), facial sensation (CN5), facial strength (CN7), hearing/balance/tinnitus (CN8), swallowing/tongue weakness (CN9-12), and other (including long tract signs and symptoms due to mass effect such as headache and proptosis). We included all symptoms at time of RT, including those which first appeared in the post-operatively. Duration of symptoms prior to RT was calculated from date of initial symptom presentation to start of RT. Durability of symptom improvement or worsening was defined as a continued subjective change or physical exam difference at the time of last followup compared to pre-RT baseline. Time to improvement and time to worsening were calculated from the start of radiotherapy to the date of change. Adverse events were defined as development of a new cranial neuropathy such as radiation induced optic neuritis (RION), trigeminal neuropathy, or other symptoms not associated with tumor progression. Radiographic findings of radiation necrosis were included as adverse events, regardless of symptoms.

### Data analysis

Statistical analysis was performed using SAS v9.2 (SAS Institute Inc, Cary, NC). Freedom from progression was estimated using Kaplan-Meier method, with differences between treatment groups calculated using the log-rank test compared to the RT alone group. Events were censored at last follow-up. We used univariate and backwards stepwise multivariate Cox proportional hazards models to estimate the hazard ratios of factors associated with tumor progression. The initial multivariate model included age, sex, prior RT, midline or bilateral location, presence of symptoms at presentation, use of IMRT, use of BrainLab planning system, prior surgery, treatment group, GTV size, fraction size (≥2 Gy vs. < 2 Gy), total dose, time from diagnosis to RT, and pathologic grade, if known. A two sided α < 0.05 was considered significant.

Univariate and backwards stepwise logistic regression model was used to determine factors associated with any symptom improvement. Covariates in the initial multivariate model included duration of symptoms prior to RT, age, gender, GTV size, fraction size, total dose, prior surgery, re-irradiation, midline or bilateral location, use of IMRT, and use of BrainLab planning system. We used logistic regression to determine the odds ratio (OR) of worsening of symptoms events in cases with progression compared to those without progression. A two sided α <0.05 was considered significant.

## Results

### Patient characteristics

This retrospective study was conducted after approval by the institutional review board at Thomas Jefferson University. We identified 278 patients treated with FSRT for skull base meningiomas from 1/1/1994 through 3/1/2009. Follow-up data were available on 217 patients (78.1%). The median follow-up time was 4.4 years. Seven patients were treated with a second course of FSRT, totaling 224 courses of FSRT included in the analysis. We considered each treatment course as a separate case for the purposes of analyses (Table 1). The majority of patients were female (74%), and the median age at the time of radiation therapy was 60.7 years old. Ninety-two percent of cases were symptomatic at the time of FSRT; the remaining cases were treated for asymptomatic progression. The most common area of involvement was the cavernous sinus (30%) or sphenoid/clinoid region (29%). These data represent approximations as tumors often encompassed multiple regions. Treatment consisted of RT alone in 51% of cases, adjuvant RT in 18%, RT for progression/recurrence after prior surgery in 26%, and re-irradiation in 5% of cases.

**Table 1:** Patient characteristics

**Variable**	**n**	**Percent**
Median Age (years)	60.7 (range 21–92)	
Median Tumor Volume (cc)	8.7 (range 0.67 – 62.2)	
Gender:		
Male	58	25.9%
Female	166	74.1%
Location:		
Cavernous sinus	66	29.5%
Parasellar	9	4.0%
Sphenoid/clinoid	65	29.0%
CPA	32	14.3%
Clivus	7	3.1%
Sellar/suprasellar	16	7.1%
Planum	15	6.7%
Craniocervical	13	5.8%
Other	1	0.4%
Laterality		
Left	95	42.4%
Right	84	37.5%
Midline/Bilateral	44	19.6%
Unknown	1	0.4%
Symptoms at time of Radiation		
Symptomatic	205	91.5%
Asymptomatic	19	8.6%
Intensity Modulate Radiation Therapy		
Yes	41	18.3%
No	183	81.7%
Neurofibramatosis-2	3	1.4%
Treatment Group*:		
Radiation only	115	51.3%
Adjuvant radiation	40	17.9%
Progression after surgery	58	25.9%
Re-irradiation	11	4.9%
Pathology		
No pathologic diagnosis	112	50.0%
WHO grade I	108	48.2%
WHO grade II	4	1.8%
Dose		
> 52.5 Gy	125	55.8%
<= 52.2 Gy	99	44.2%

* Per treatment course. Seven patients in the radiation only, adjuvant radiation or progression after surgery groups are also present in the re-irradiation group because they underwent a second course of fractionated stereotactic radiation therapy.

### Local control

**Figure 1:**
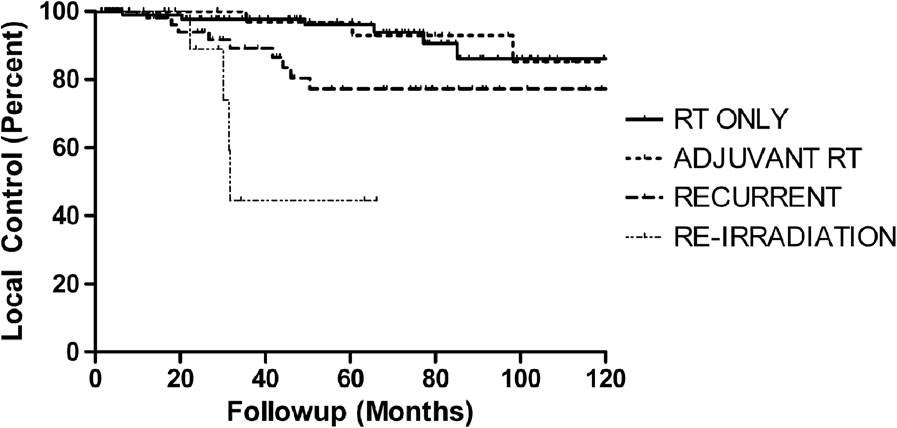
**Kaplan meier local control by treatment group.** Seven patients in the re-irradiation arm are also counted in one other arm because of recurrence. Compared to radiation therapy (RT) alone, adjuvant RT was not different (p=0.98), but recurrence after prior surgery (p = 0.02) and re-irradiation (with or without prior surgery) groups (p<0.01) were different.

Twenty-one patients progressed after FSRT, representing a 90.6% cumulative freedom from local failure. Local control at 5 years was 96.1% in the RT alone group, 96.8% (p=0.75) in the post-operative group, 77.3% (p=0.04) in the progression/recurrence after surgery group, and 44.4% (p < 0.01) in the progression after radiation group (Figure 1). In addition to treatment group, recurrence after prior RT (Hazard ratio (HR) 8.4, p<0.01), midline/bilateral tumor location (HR 2.9, p=0.02), any prior surgery (HR 2.51, p=0.05), treatment volume per each cm [3] larger (HR 1.032, p=0.03) and use of IMRT (HR 2.73, p=0.04), were significantly correlated with increased rate of local failure on univariate analysis. Total dose, use of BrainLab planning system, being symptomatic at presentation and fraction size were not correlated with local failure. On multivariate analysis, midline/bilateral location (HR 2.97, p=0.02), atypical histology (HR 8.70, p=0.01) and treatment group were associated with local failure. Compared to RT alone, adjuvant RT (HR 0.82, p=0.79) and RT for recurrence after surgery (HR 1.78, p=0.32) were not significantly different; however, re-irradiation (HR 11.02, p<0.01) was a significant predictor of subsequent local failure.

### Symptom presentation

Patients were symptomatic at the start of treatment in 92% of cases. The most common presenting symptoms were decreased visual acuity/visual field deficits (57.6%) and diplopia/ptosis (33.5%) (Table 2). In our cohort, the frequency of patients who received surgery prior to RT differed in each symptom category. Approximately 40% of cases presenting with diplopia or ptosis had surgery prior to RT, while 90% of those presenting with facial weakness or CN9-12 deficits had surgery prior to RT. Symptoms were first noted in the post-operative setting in several cases, including facial weakness (50%), CN9-12 deficits (37.5%) and decreased facial sensation (18%). The median duration of symptoms prior to radiation was generally around 1 year (Table 2), and was longer in cases with prior surgery (data not shown).

**Table 2:** Distribution of presenting symptoms at the time of radiation therapy

**Domain**	**Number of cases**	Percent of all cases*	Median duration (months)**	**Prior surgery**	New symptom post-op***
Visual Acuity/Visual Field	129	57.6%	12.8	51.9%	0.8%
Any Diplopia/Ptosis	75	33.5%	9.9	41.3%	2.7%
Cranial Nerve III	37	16.5%			
Cranial Nerve IV	8	3.6%			
Cranial Nerve VI	39	17.4%			
Unspecified	20	8.9%			
Facial Sensation	50	22.3%	9.9	50.0%	18.0%
Facial Weakness	12	5.4%	8.4	91.7%	50.0%
Hearing	34	15.2%	13.6	50.0%	17.6%
Balance	32	14.3%	12.9	43.8%	6.3%
Tinnitus	7	3.1%	26.4	28.6%	0
Cranial Nerve IX -XII	8	3.6%	14.7	100%	37.5%
Other	61	27.2%	9.7	45.9%	3.3%
Asymptomatic	19	8.5%	--	47.4%	--

* Do not add up to 100% because some patient presented with multiple symptoms.

**Duration of symptom prior to radiation therapy.

***Among cases with the specific deficit at radiation, percent where the symptom only appeared in the post-operative setting.

### Symptom outcomes

**Figure 2:**
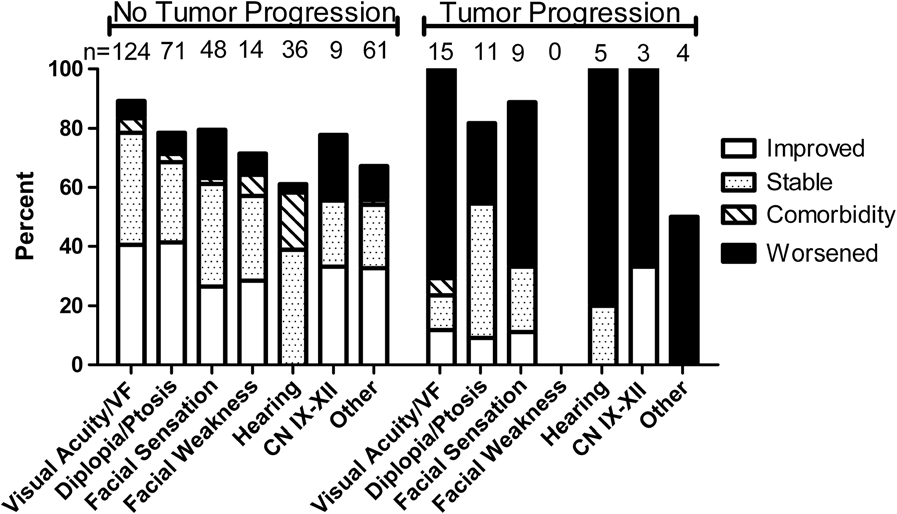
**Symptom outcome after FSRT in cases without progression and with progression.** Symptom outcomes (improved, stable, comorbidity, stable) refer to the final status of the symptom at last followup. Comorbidity refers to symptom worsening due to a co-morbid disease process and unrelated to tumor progression or treatment. The number of cases is greater than number who presented with each symptom because some patients developed new deficits due to worsening of symptoms from either tumor progression, treatment toxicity or co-morbid disease.

Symptom outcomes varied according to initial symptoms (Figure 2). The outcomes of 10-30% of symptoms after FSRT were not documented, particularly for those related to hearing and balance. Among cases without tumor progression, 30-40% had durable improvement, 30-40% had stable symptoms, and less than 10% had symptom deterioration after FSRT (Figure 2). The most common presenting symptom, visual acuity and visual field deficits, was durably improved in 37% of cases, maintained in 35%, and deteriorated after tumor progression or treatment related adverse event in 14%. Additionally several patients developed new symptoms attributable to co-morbid conditions, such as worsening vision in a patient with HSV keratitis or new deficits following a stroke. Durable symptom improvement of at least one symptom occurred in 57% of cases.

**Figure 3:**
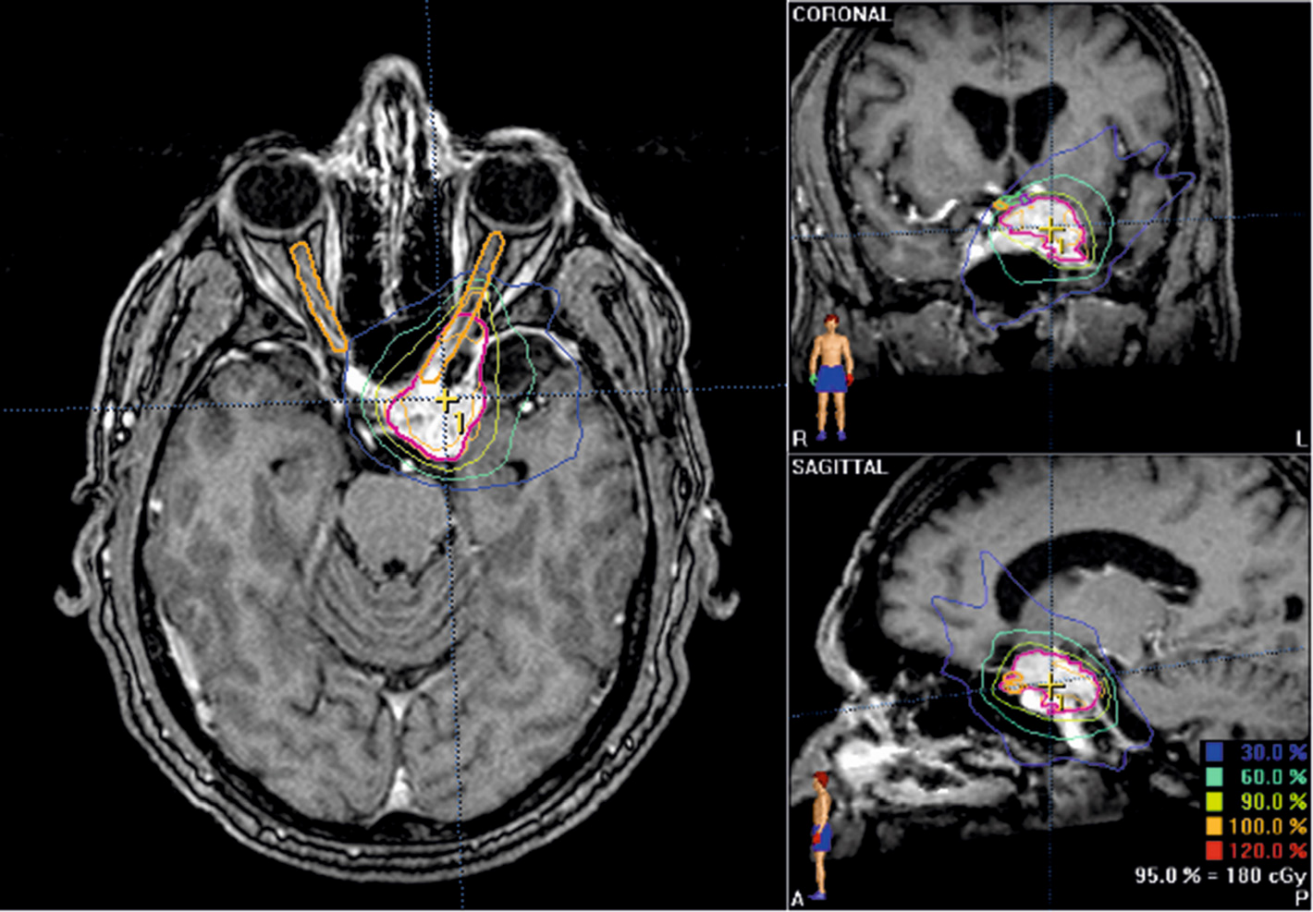
**Example of plan from case of very early visual acuity improvement during FSRT course.** Patient was treated to 52.2 Gy at 1.8 Gy/fraction to the 95% iso-dose line using an eight field stereotactic fractionated intensity modulated radiation therapy plan.

Symptom improvement typically occurred within 6 months of the start of FSRT (Table 3), and often earlier. By the last day of FSRT course, 17% of cases had experienced improvement in at least one symptom, and by 2 months after FSRT, 27% of cases had improvement in at least one symptom. Of cases with symptom improvement, 33% first occurred by the end of FSRT and 54% within 2 months after FSRT. In contrast, symptom deterioration was observed many months later (median 18.7 months). In the absence of tumor progression, 89% – 100% of symptom improvements were durable at the time of last follow-up. For an illustrative example of early symptom improvement (Table 4 and Figure 3).

**Table 3:** Timing and durability of symptom improvement

**Domain**	**Median time to improve (mo.)**	**Range (mo.)**	Durability of improve*	**Median time to worsening (mo.) (range)**	**Range (mo.)**
Visual Acuity/Field	3.2	0.1 - 30.1	92.7%	25.0	0.2 - 98.2
Diplopia/Ptosis	4.6	0.6 - 128.7	88.2%	47.0	0.5 - 85.9
Facial Sensation	4.8	0.6 - 58.6	93.3%	17.3	2.5 - 102.7
Facial Weakness	16.8	1.1 - 42.8	100%	33.3	1.9 - 64.9
Hearing	--	2.1 - 2.7	--	5.1	2.5 - 82.8
Balance	2.4	2.8 - 2.8	100%	11.1	2.8 - 114.0
Tinnitus	2.8		100%	3.5	2.4 - 4.6
Cranial Nerve IX-XII	4.9	4.2 - 5.6	100%	24.6	6.4 - 31.5
Other	2.4	0.5 - 5.6	91.7%	9.5	2.4 - 20.3

* Durability defined as percent of symptoms which after initial improvement remained improved over pre-radiation baseline at the time of last follow-up.

**Table 4:** Example of very early visual acuity improvement during fractionated stereotactic radiation therapy course

**Date**	**Visit reason**	**Time from start of RT (months)**	**Visual acuity left eye**	Humphrey visual field (MSD/PD*) left eye
2/21/2006	Initial Consult	--	20/200+	−26.52/6.96
4/7/2006	On treatment (27 Gy)	0.6	20/100	
4/12/2006	On treatment (32.4 Gy)	0.8	20/50	
6/1/2006	Follow-up	2.4	20/30	−7.51/7.25
2/8/2007	Follow-up	11	20/25	−5.21/4.81
2/14/2008	Follow-up	23	20/25	
11/18/2009	Follow-up	44	20/25	−5.76/7.60

*Mean standard deviation/Percent deviation.

Patient M.R. initially presented with left visual field deficits and decreased visual acuity in 1996 and underwent a subtotal resection of a left cavernous sinus meningioma with improvement. He had symptomatic worsening in 2005, and a radiographic evidence of progression in January 2006. He received FSRT from 3/20/2006 to 4/27/2006 reporting subjective improvement in vision in the third week of fractionated stereotactic radiation therapy, confirmed objectively on physical examination by Snellen chart using a near card. Objective improvement was also confirmed on Humphrey visual field testing. His vision remained excellent through last follow-up.

### Symptoms and progression

Symptom worsening occurred more often in cases where tumors progressed (68% vs. 13%, p < 0.01). Patients with tumor progression had a 12 fold increased odds of symptoms worsening (OR 12.29 95% CI 4.6 – 32.6, p < 0.01). The most common presenting symptom, visual field/visual acuity deficit, worsened in 12 of 17 (70.6%) patients who ultimately experienced progression. Worsening of symptoms commonly preceded objective radiographic finding of tumor progression (data not shown).

### Predictors of symptom improvement

On univariate analysis, prior surgery (OR 0.44, 95% CI 0.25-0.78), midline/bilateral location of tumor (OR 0.44, 95% CI 0.22-0.90), and re-irradiation (OR 0.18, 95% CI 0.38-0.88) were negative predictors of symptomatic improvement after FSRT. Of note, GTV size, fraction size, total dose, use of IMRT, use of BrainLab planning system, and duration of symptoms prior to FSRT were not associated with symptom improvement. On multivariate analysis, any prior surgery (OR 0.47, 95% CI 0.25-0.86), and age (per 10 years older) (OR 0.78, 95% CI 0.62-0.98) were negative predictors of symptom improvement, while re-irradiation (OR 0.40, 95% CI 0.11 – 1.40), midline/bilateral location was no longer significant (OR 0.53, 95% CI 0.25-1.11) (Table 5).

**Table 5:** Multivariate predictors of symptom improvement

**Predictor (Multivariate)**	**Odds ratio**	**95%CI**	**p-value**
Age (per 10 years older)	0.78	0.62 – 0.98	0.03
Prior Surgery*	0.47	0.25 – 0.86	0.01
Re-irradiation	0.40	0.11 – 1.40	0.15
Midline/Bilateral location	0.53	0.25 – 1.11	0.09

Symptom improvement defined as improvement of at least one symptom compared to prior to radiation therapy. Multivariate model based on backwards stepwise logistic regression.

*Patients who either received adjuvant radiation or salvage radiation after prior surgery alone.

### Adverse outcomes

We noted 32 adverse events in 28 patients (12.5% cumulative incidence). The most common adverse events were radiation induced optic neuritis (n = 5, 2.2%), radiation necrosis (n = 5, 2.2%) and facila numbness or pain (n = 5, 2.2%). Additional adverse events included three cases of hypothyroidism, two cases each of diplopia, significant cognitive decline, and occipital neuralgia, and one case each of dysphagia, facial myoclonus, glossopharyngeal neuralgia, seizures,, diabetes insipitus, and vertigo.

## Discussion

We present the results of our institutional experience with FSRT for the treatment of skull base meningiomas. Our local control outcomes are consistent with other reports of 5 year progression free survival rates of 87–95.7% after treatment with either SRS [11-13,15] or FSRT [8-11]. Since many of these patients are symptomatic at presentation (92% in our cohort) we feel that symptom outcome is a crucial end-point in addition to local control and overall survival, which tend to be universally excellent in published reports. This report adds to the existing literature on by providing a detailed analysis dissected by symptom domain in a large retrospective cohort. These patients were closely followed during RT, allowing detection of a high rate of symptom improvement early in the treatment course.

We found that in addition to providing excellent local control, FSRT resulted in durable symptom improvement of one or more symptoms in 57% of cases. For patients with reduced vision, FSRT durably improved or maintained vision in at least 71% of cases, demonstrating the utility of FSRT in function preservation. While less detailed, other series in the literature have also reported between a 5-46% rate of improvement in symptoms following radiation therapy with either SRS or FSRT [8,9,15,16]. We have not attempted to compare these results with those reported from SRS or surgical series because =of multiple potential confounding factors such as case mix, recall bias and reporting bias.

We found a negative association between prior surgery, re-irradiation and increasing age with the likelihood of symptom improvement after FSRT. These finding suggest FSRT after prior definitive treatment is less likely to result in symptomatic improvements, particularly in older patients. Midline or bilateral location was a negative prognostic factor for symptom improvement, a finding which has not been reported previously. Midline and bilateral tumors were larger, received less radiation dose, and underwent resection prior to radiation more often. It is unclear whether this finding is the result of an intrinsic property of tumors of this region or a spurious finding as a result of multiple comparisons, and should be investigated in future studies. Of interest, despite evolving technology, the use of the BrainLab treatment planning system, a surrogate for patients treated prior to 2004 versus those treated after 2004, predict for either local control or symptom improvement.

When patient symptoms improved, they did so by the end of FSRT in one third of patients, and within two months of the end of FSRT in over half of patients. This observation of the early symptom improvement raises questions regarding the underlying mechanism. Such improvement was also reported in the literature to occur at 6 weeks after completion of IMRT [17] and FSRT [18]. This time frame typically precedes radiographic shrinkage and likely occurs before the full tumoricidal effect of radiation. A placebo effect is unlikely to account for this symptoms relief, as patients were noted to have objective responses on cranial nerve examinations during weekly treatment visits. Although speculative, one possible explanation for restoration of cranial nerve function after radiation is re-distribution of vascular flow from tumor to the affected cranial nerve(s).

Conversely, while radiographic improvement does not necessarily correlate with symptomatic improvement, there was a strong relationship between radiographic tumor progression and symptom worsening. The mechanism of symptom worsening may differ from that of improvement. Indeed, the clinical finding of a new symptom may be an early indicator of radiographic progression.

Treatment was generally very well tolerated and adverse events were infrequent in our series, though estimates were conservative and included all possible treatment related events. Our reported rates of RION (2.2%) and radiation necrosis (2.2%) were low and may still over-estimate the actual adverse event rates, supporting FSRT as a safe treatment option.

Limitations to our study include limited documentation of symptom severity and relatively short median follow-up time. Although we recorded symptom severity, these were often recorded as subjective rather than objective measures. Some of patients did have objective measurement of symptom outcomes, such as with visual field testing, and these have typically correlated well with objective findings. We have chosen to not report the objective findings in this report as they represent only a subset of the overall cohort and may not be representative of overall outcomes. Additionally, given the difficulty in comparing severity between symptom domains, we did not use this measure in analysis.

The median follow-up time (4.4 years) is short given the range of patients who were treated from 1994 through 2009. Factors for this finding include losses to follow-up from patients who live far from our facility and to storage from paper records. Additionally, we strictly defined follow-up as the last evaluation by a radiation oncologist or a neurosurgeon, rather than the last evidence of patient being alive, in order to capture symptom outcomes. A quarter of our patients had follow-up greater than 5.8 years, permitting a limited evaluation of long term toxicity and symptom outcomes.

## Conclusions

Fractionated stereotactic radiation therapy offers a safe and effective treatment option for patients with skull base meningiomas. Over half of patients experience durable improvement in one or more symptoms, often within a few months following the start of radiation therapy. Our results support FSRT as a safe and effective treatment for patients with base of skull meningiomas.

## Abbreviations

CN: Cranial nerve; CTV: Clinical target volume; GTV: Gross tumor volume; FSRT: Fractionated stereotactic radiation therapy; HR: Hazard ratio; IMRT: Intensity modulated radiation therapy; LINAC: Linear accelerator; OR: Odds ratio; PTV: Planning target volume; RT: Radiation therapy; RION: Radiation induced optic neuritis; SRS: Stereotactic radiosurgery.

## Competing interests

The authors declare that they have no competing interests.

## Authors’ contributions

XS: concept design, data acquisition, data analysis, drafting of manuscript, revisions, DA: concept design, data acquisition, revision of manuscript, RS: data acquisition, JE: data acquisition, WC: data acquisition, MM: data acquisition, revision of manuscript, RF: concept design, data acquisition, HE: data acquisition, CC: data acquisition, MW: data acquisition, MVM: data analysis, drafting of manuscript, revision of manuscript, AD: data analysis, MW-W: concept design, data acquisition, drafting of manuscript, revision of manuscript. All authors read and approved the final manuscript.
